# Supplementary material for: Identification of HDV-like theta ribozymes involved in tRNA-based recoding of gut bacteriophages
Source: Nat Commun. 2024 Feb 20;15:1559. doi: 10.1038/s41467-024-45653-w (PMC10879173; doi:10.1038/s41467-024-45653-w)
Supplement: Supplementary file 1 — Supplementary Information [file 41467_2024_45653_MOESM1_ESM.pdf]

Supplementary Information for

Identification of HDV-Like *Theta* Ribozymes Involved in tRNA-based Recoding of Gut Bacteriophages

Author list

Kasimir Kienbeck[1]†, Lukas Malfertheiner[2]†, Susann Zelger-Paulus[1], Silke Johannsen[1], Christian von Mering[2]*, Roland K.O. Sigel[1]*

Affiliations

[1]Department of Chemistry, University of Zurich; Zurich, CH-8057, Switzerland.

[2]Department of Molecular Life Sciences and Swiss Institute of Bioinformatics, University of Zurich; Zurich, CH-8057, Switzerland.

*Corresponding authors. Email: christian.von.mering@mls.uzh.ch, roland.sigel@chem.uzh.ch

†These authors contributed equally to this work.

The file includes:

Supplementary Figs. 1 to 7

Supplementary Tables 1 to 3

Other Supplementary Materials for this manuscript include the following:

Supplementary Data Files 1 to 6

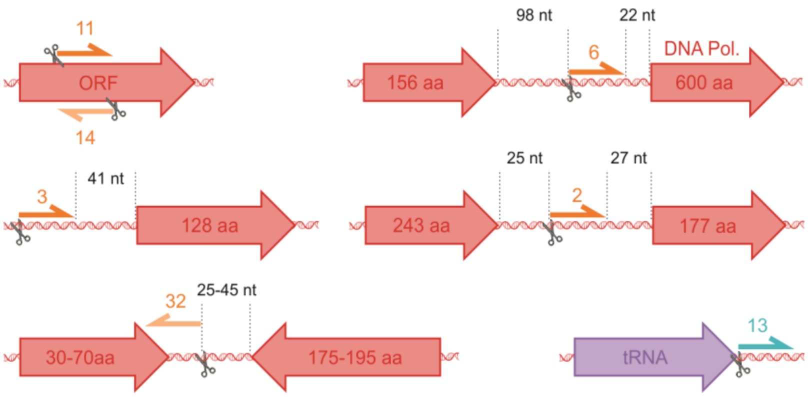

Supplementary Fig. 1 | Initial classification of minimal drzs. Minimal drzs (orange half-arrows and numbers; orientation from 5' to 3') discovered in annotated phage genome databases[1,2] using the search motif shown in Fig. 2b (i). Classification with respect to nearby annotated viral open reading frames (ORF; red arrows). DNA polymerase encoding ORF: DNA Pol.; Self-scission site of drzs indicated by scissors; distance to ORFs indicated in number of nucleotides (nt; black); ORF lengths indicated in number of amino acids (aa); viral tRNA encoding sequence: purple; tRNA-associated drzs: turquoise; drzs in the same orientation as ORFs (sense) depicted in dark colors; drzs in the opposite orientation as ORFs (antisense) depicted in light colors.

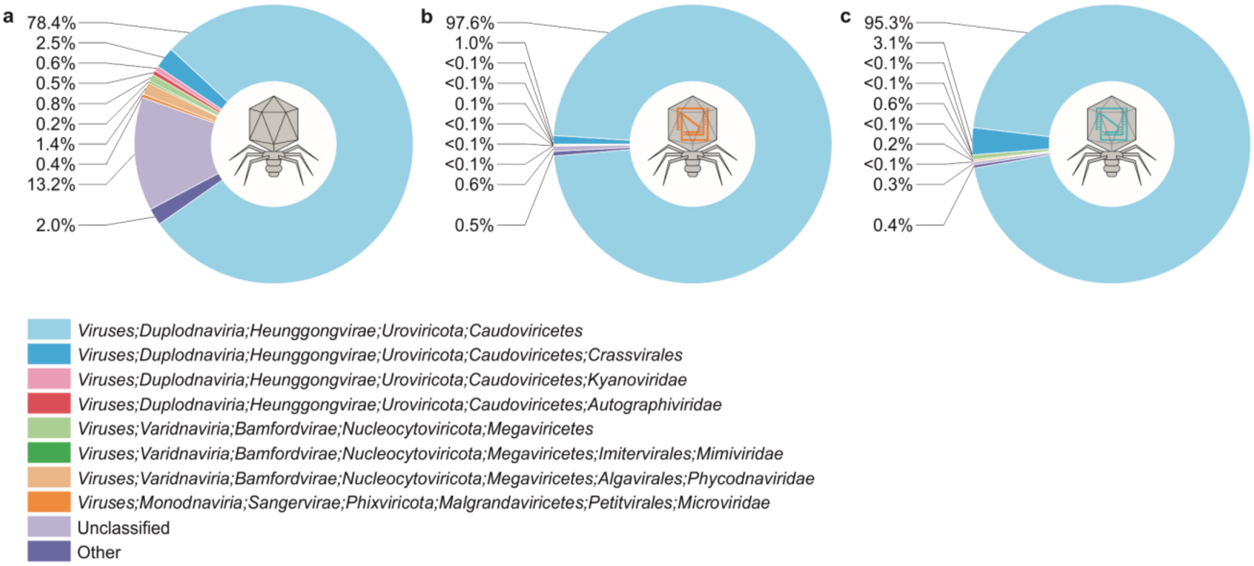

Supplementary Fig. 2 | Phage taxonomy. a, Taxonomy of all viruses that were searched. **b**, Taxonomy of viruses containing at least one minimal drz resulting from the initial search motif shown in Fig. 2b (i). **c**, Taxonomy of viruses containing at least one Θrz resulting from the final search motif shown in Fig. 2b (iv). Almost all viruses (>98%) with an identified Θrz belong to the *Caudoviricetes*. The taxonomy of all phage genomes was determined using geNomad[3].

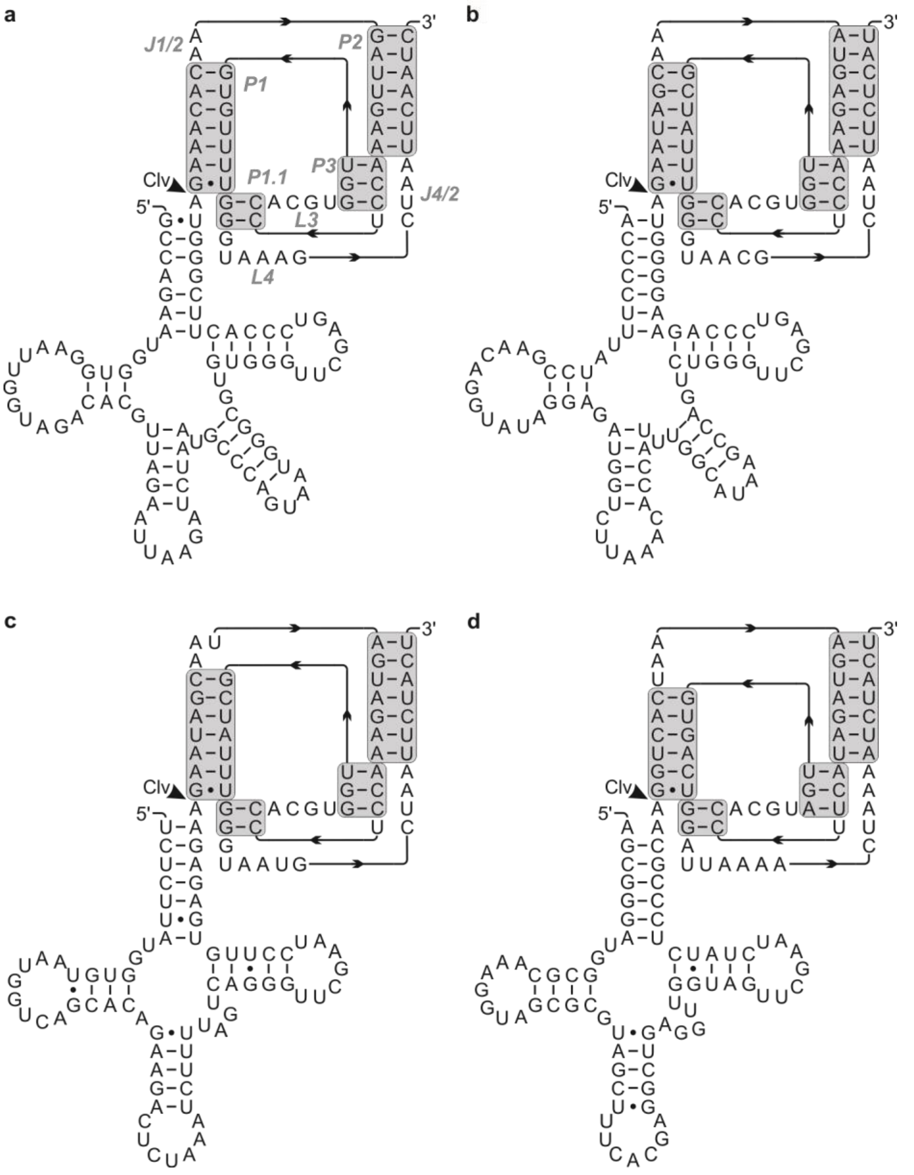

Supplementary Fig. 3 | Secondary structures of analyzed tRNA/Θrz pairs. a, Proposed secondary structure of tRNA^{Leu}0024_Θ0009. Helical domains of the Θrz are highlighted in gray. Ribozyme domain labels in gray italics. **b**, Proposed secondary structure of tRNA^{Leu}0112_Θ0016, labeling as in **a**. **c**, Proposed secondary structure of tRNA^{Sup}0028_Θ0092, labeling as in **a**. **d**, Proposed secondary structure of tRNA^{Val}0025_Θ0046, labeling as in **a**. The cleavage site (Clv) is indicated by an arrowhead.

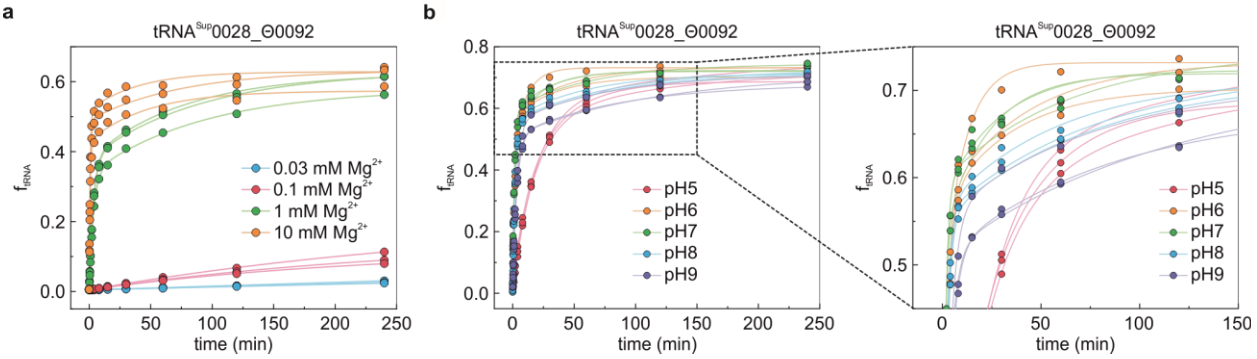

Supplementary Fig. 4 | Representative fits of tRNA^{Sup}0028_Θ0092 self-cleavage. a, f_{tRNA} dependency on Mg^{2+} calculated with Eq. 1. The curve was fitted using Eqs. 2 and 3. **b**, f_{tRNA} dependency on pH calculated with Eq. 1 and enlargement for clarity. The curve was fitted using Eqs. 2 and 3.

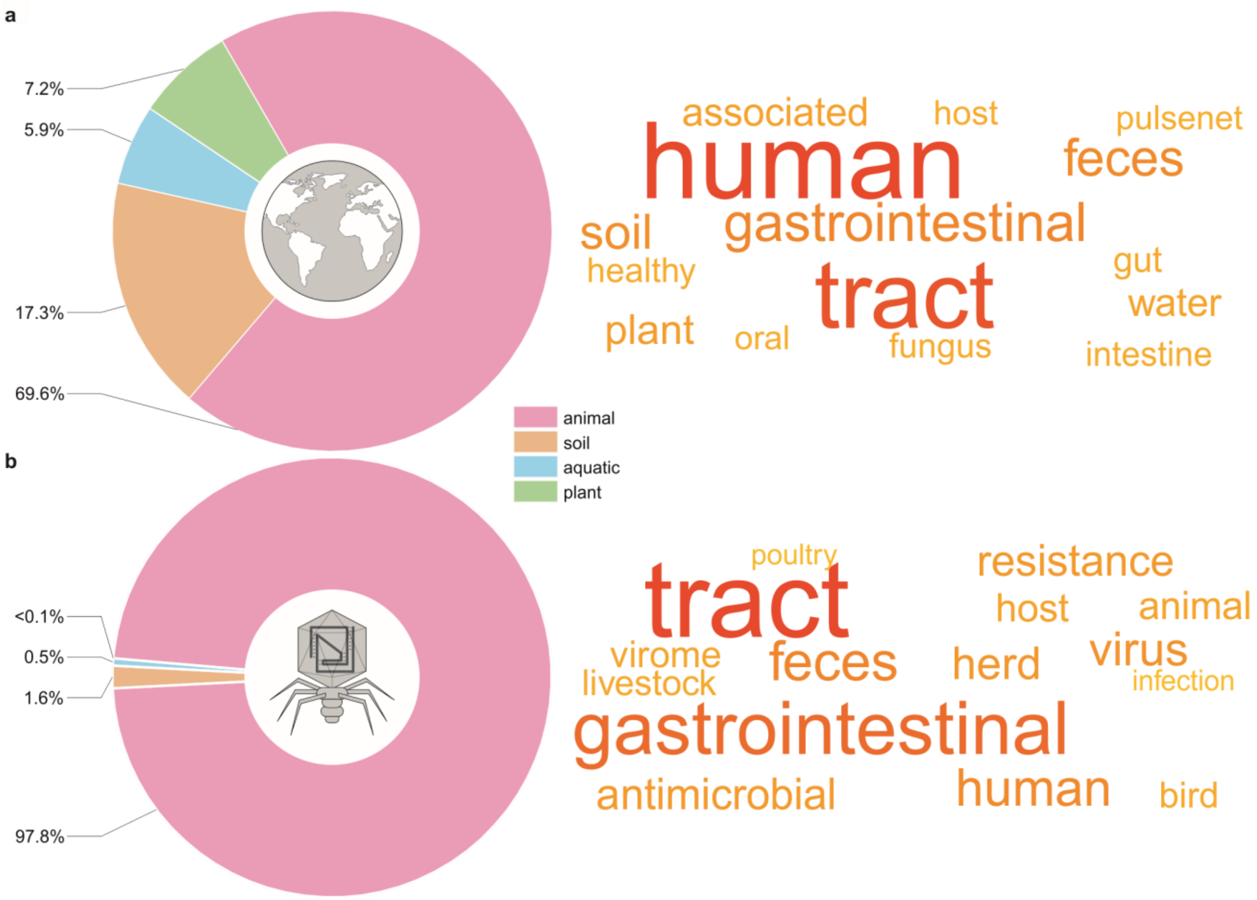

Supplementary Fig. 5 | Animal gut is the main habitat for Θrzs. Environments and keywords from annotated samples within the Microbe Atlas Project. The darkness and size of the words correlate with their occurrences (darkest, largest = most common). **a**, Background distribution of keywords and environments of metagenomic samples within the Microbe Atlas Project: 10,000 samples were randomly selected, and the 15 most common keywords are shown. **b**, The 15 most common keywords and environments from all samples in which tRNA-associated Θrzs are detected. Keywords related to viruses that were absent in the background distribution are now the top hits, whereas keywords such as "water", "plant" or "soil" are absent. The resulting word clouds point to viruses in the animal gut as the predominant Θrz environment.

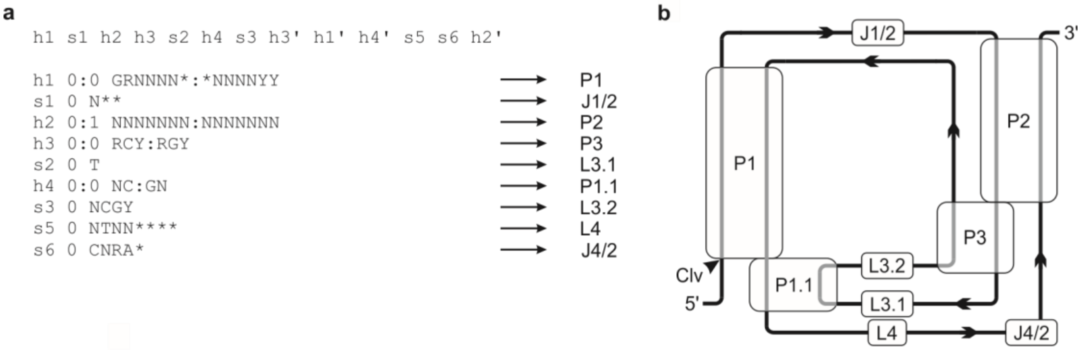

Supplementary Fig. 6 | Structure of the refined Θrz descriptor file. a, Structure of the descriptor file of the final motif used in the RNArobo[4] motif search with corresponding domain names of the minimal drz motif. **b**, Graphical illustration of minimal drz secondary structure and domain names for reference.

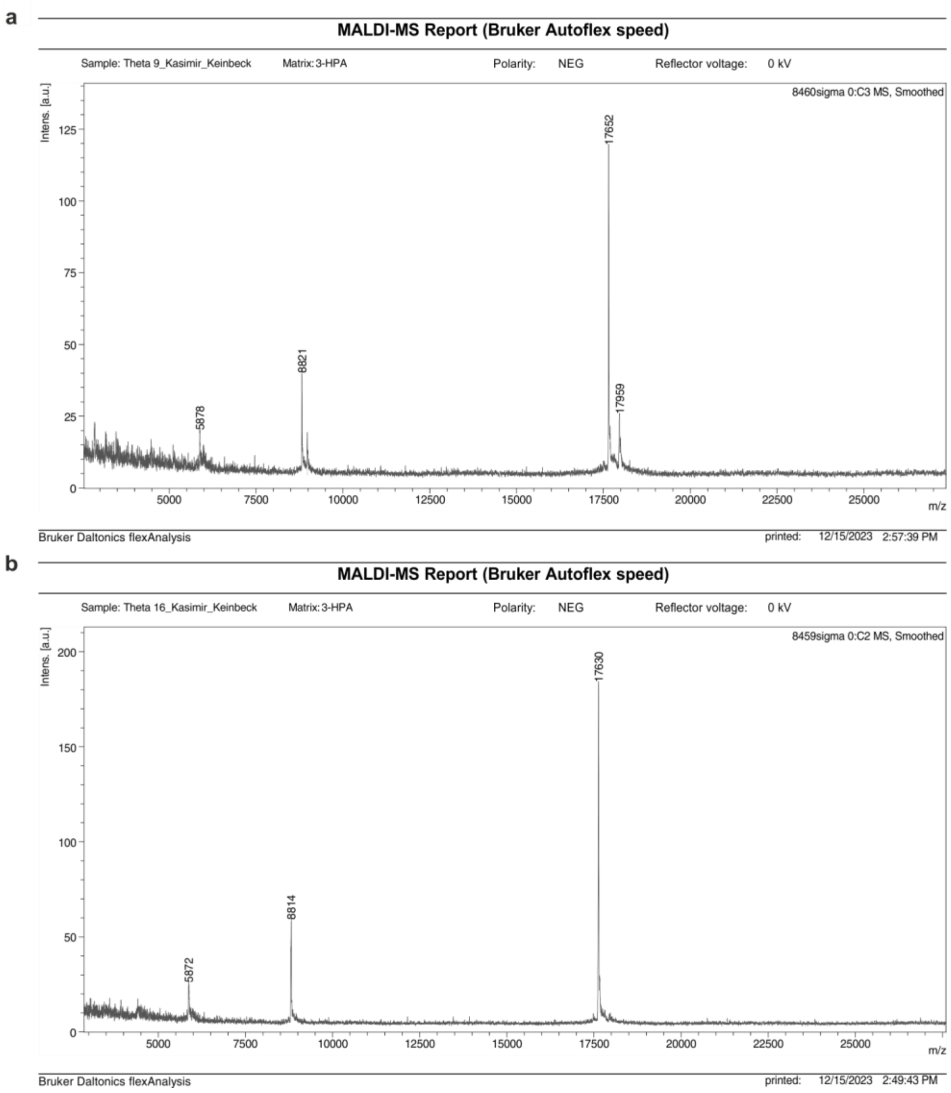

Supplementary Fig. 7 | MALDI-TOF-MS of two co-transcriptionally cleaved Θrz examples. **a**, MALDI-TOF-MS of Θrz0009 (calculated mass: 17.65 kDa). The additional, smaller peak corresponds to the n+1 band from (run-off) *in vitro* transcription (also observable on the gels), with an additional C or U added (+ 0.308 kDa; calculated mass: 17.96 kDa). **b**, MALDI-TOF-MS of Θrz0016 (calculated mass: 17.63 kDa). To the left in both spectra, the peaks of m/z with z=2 and z=3 show up. All masses were calculated with a 5'-OH group due to co-transcriptional cleavage of the ribozyme.

Supplementary Table 1 | Summary of the investigated databases in the search for Θrzs. Amount of detected Θrzs and tRNA-associated Θrzs in eight different bacteriophage databases. Representative genomes from proGenomes3 and eukaryotic genomes are also shown at the bottom.

name of database	total no. of isolated Θrzs	total no. of tRNA-associated Θrzs	total no. of analyzed bp (Gbp)	origin
Tisza, 2021[2]	88	58	6.4	human gut
Camarillo-Guerrero, 2021[5]	342	241	10.7	human gut
Nayfach, 2021[6]	565	364	17.6	human gut
Borges, 2022[7]	26	16	0.1	animal gut
Li, 2022[8]	2	2	2.9	human oral
Gregory, 2019[9]	7	0	7.2	marine
Stano, 2016[10]	0	0	0.8	all environments
Roux, 2021[11]	239	60	97.1	all environments
proGenomes3[12]*	12	0	310.6	bacterial genomes
NCBI RefSeq[13]*	0	0	7.8	eukaryotic genomes

*non-viral databases

Supplementary Table 2 | Apparent Mg^{2+}-dependent cleavage rate constants (k_{obs}) of selected tRNA/Θrz pairs.

[Mg^{2+}] = Magnesium(II) concentration (mmol/L); k_1 = apparent cleavage rate constant (min^{-1}); rep1-3 = replicate 1-3; mean(k_1) = mean value of k_1 (min^{-1}); σ = standard deviation of the mean (min^{-1}); k_{max} = maximal apparent cleavage rate constant (min^{-1}); SE = standard error of the fit (min^{-1}); K_d = dissociation constant (mM); n = Hill coefficient.

				**$tRNA^{Leu}$0024_Θ0009**			
[Mg^{2+}]	k_1(rep1)	k_1(rep2)	k_1(rep3)	mean(k_1) ± 3σ	k_{max} ± SE	K_d ± SE	n ± SE
0.03	0.0028	0.0029	0.0024	0.0027 ± 0.0007	1.11 ± 0.27	1.51 ± 0.60	1.75 ± 0.21
0.1	0.010	0.006	0.008	0.008 ± 0.007			
1	0.48	0.52	0.49	0.50 ± 0.07			
10	0.94	1.02	1.09	1.02 ± 0.22			
				**$tRNA^{Leu}$0112_Θ0016**			
[Mg^{2+}]	k_1(rep1)	k_1(rep2)	k_1(rep3)	mean(k_1) ± 3σ	k_{max} ± SE	K_d ± SE	n ± SE
0.03	0.0054	0.0063	0.0065	0.0061 ± 0.0019	2.32 ± 0.43	0.84 ± 0.27	1.88 ± 0.22
0.1	0.030	0.036	0.037	0.034 ± 0.011			
1	1.94	2.58	2.59	2.37 ± 1.13			
10	1.64	2.44	2.61	2.23 ± 1.55			
				**$tRNA^{Sup}$0028_Θ0092**			
[Mg^{2+}]	k_1(rep1)	k_1(rep2)	k_1(rep3)	mean(k_1) ± 3σ	k_{max} ± SE	K_d ± SE	n ± SE
0.03	0.00011	0.00009	0.00008	0.00009 ± 0.00004	1.20 ± 0.22	1.53 ± 0.23	2.38 ± 0.08
0.1	0.0029	0.0036	0.0043	0.0036 ± 0.0020			
1	0.30	0.29	0.29	0.29 ± 0.02			
10	1.28	1.18	1.22	1.22 ± 0.15			
				**$tRNA^{Val}$0025_Θ0046**			
[Mg^{2+}]	k_1(rep1)	k_1(rep2)	k_1(rep3)	mean(k_1) ± 3σ	k_{max} ± SE	K_d ± SE	n ± SE
0.03	0.00006	0.00007	0.00020	0.00011 ± 0.00023	>10	-	-
0.1	0.0006	0.0006	0.0019	0.0010 ± 0.0023			
1	0.58	0.45	1.05	0.69 ± 0.94			
10	>10	>10	>10	>10			

Supplementary Table 3 | Apparent pH-dependent cleavage rate constants (k_{obs}) of selected tRNA/Θrz pairs.

[Mg^{2+}] = Magnesium(II) concentration (mmol/L); k_1 = apparent cleavage rate constant (min^{-1}); rep1-3 = replicate 1-3; mean(k_1) = mean value of k_1 (min^{-1}); σ = standard deviation of the mean (min^{-1}); k_{max} = maximal apparent cleavage rate constant at 0.5 mM Mg^{2+} (min^{-1}); SE = standard error of the fit (min^{-1}); pK_{a1} = pK_a of the hydrated Mg^{2+} ion; pK_{a2} = pK_a of the catalytic cytosine residue.

**tRNA^{Leu}0112_Θ0016**							
pH	k_1(rep1)	k_1(rep2)	k_1(rep3)	mean(k_1) ± 3σ	k_{max} ± SE	pK_{a1} ± SE	pK_{a2} ± SE
5.06	0.0073	0.0076	0.0074	0.0074 ± 0.0005			
6.05	0.25	0.26	0.31	0.27 ± 0.10			
6.97	0.73	0.76	0.78	0.75 ± 0.07	0.88 ± 0.03	9.0 ± 0.1	6.3 ± 0.1
8.01	0.72	0.73	0.74	0.73 ± 0.04			
9.01	0.43	0.44	0.44	0.44 ± 0.03			
**tRNA^{Sup}0028_Θ0092**							
pH	k_1(rep1)	k_1(rep2)	k_1(rep3)	mean(k_1) ± 3σ	k_{max} ± SE	pK_{a1} ± SE	pK_{a2} ± SE
5.06	0.052	0.086	0.055	0.064 ± 0.056			
6.05	0.64	0.44	0.42	0.50 ± 0.37			
6.97	0.76	0.83	0.69	0.76 ± 0.21	0.75 ± 0.06	8.7 ± 0.2	5.8 ± 0.2
8.01	0.49	0.49	0.58	0.52 ± 0.15			
9.01	0.30	0.29	0.31	0.30 ± 0.04			
**tRNA^{Val}0025_Θ0046**							
pH	k_1(rep1)	k_1(rep2)	k_1(rep3)	mean(k_1) ± 3σ	k_{max} ± SE	pK_{a1} ± SE	pK_{a2} ± SE
5.06	0.0079	0.0068	0.0073	0.0073 ± 0.0017			
6.05	0.032	0.030	0.030	0.031 ± 0.003			
6.97	0.079	0.080	0.074	0.078 ± 0.009	0.084 ± 0.003	9.0 ± 0.1	6.2 ± 0.1
8.01	0.070	0.070	0.067	0.069 ± 0.005			
9.01	0.044	0.041	0.043	0.043 ± 0.005			

References

1. Nishijima, S. *et al.* Extensive gut virome variation and its associations with host and environmental factors in a population-level cohort. *Nat. Commun.* **13**, 5252 (2022).
2. Tisza, M. J. & Buck, C. B. A catalog of tens of thousands of viruses from human metagenomes reveals hidden associations with chronic diseases. *Proc. Natl. Acad. Sci.* **118**, e2023202118 (2021).
3. Camargo, A. P. *et al.* Identification of mobile genetic elements with geNomad. *Nat. Biotechnol.* 1–10 (2023) doi:10.1038/s41587-023-01953-y.
4. Rampášek, L., Jimenez, R. M., Lupták, A., Vinař, T. & Brejová, B. RNA motif search with data-driven element ordering. *BMC Bioinformatics* **17**, 216 (2016).
5. Camarillo-Guerrero, L. F., Almeida, A., Rangel-Pineros, G., Finn, R. D. & Lawley, T. D. Massive expansion of human gut bacteriophage diversity. *Cell* **184**, 1098–1109 (2021).
6. Nayfach, S. *et al.* Metagenomic compendium of 189,680 DNA viruses from the human gut microbiome. *Nat. Microbiol.* **6**, 960–970 (2021).
7. Borges, A. L. *et al.* Widespread stop-codon recoding in bacteriophages may regulate translation of lytic genes. *Nat. Microbiol.* **7**, 918–927 (2022).
8. Li, S. *et al.* A catalog of 48,425 nonredundant viruses from oral metagenomes expands the horizon of the human oral virome. *iScience* **25**, 104418 (2022).
9. Gregory, A. C. *et al.* Marine DNA Viral Macro- and Microdiversity from Pole to Pole. *Cell* **177**, 1109–1123 (2019).
10. Stano, M., Beke, G. & Klucar, L. viruSITE-integrated database for viral genomics. *Database J. Biol. Databases Curation* **2016**, baw162 (2016).
11. Roux, S. *et al.* IMG/VR v3: an integrated ecological and evolutionary framework for interrogating genomes of uncultivated viruses. *Nucleic Acids Res.* **49**, D764–D775 (2021).

12. Fullam, A. *et al.* proGenomes3: approaching one million accurately and consistently annotated high-quality prokaryotic genomes. *Nucleic Acids Res.* **51**, D760–D766 (2023).

13. O'Leary, N. A. *et al.* Reference sequence (RefSeq) database at NCBI: current status, taxonomic expansion, and functional annotation. *Nucleic Acids Res.* **44**, D733–D745 (2016).
