# Supplementary material for: Identification of HDV-like theta ribozymes involved in tRNA-based recoding of gut bacteriophages
Source: Nat Commun. 2024 Feb 20;15:1559. doi: 10.1038/s41467-024-45653-w (PMC10879173; doi:10.1038/s41467-024-45653-w)
Supplement: Supplementary file 3 — Description of Additional Supplementary Files [file 41467_2024_45653_MOESM3_ESM.docx]

**Description of Additional Supplementary Files**

**File Name: Supplementary Data 1**

**Description:** Table containing the combined tRNA-associated Θrz sequences from annotated and metagenomic samples sorted by Θrz frequency in descending order. Contains tRNA-associated Θrzs, the respective tRNA sequences, and additional information such as the origin, taxonomy, and predicted hosts, where available.

**File Name: Supplementary Data 2**

**Description:** Table containing the combined isolated Θrz sequences from annotated and metagenomic samples sorted by Θrz frequency in descending order. Contains non-tRNA-associated Θrz sequences and additional information.

**File Name: Supplementary Data 3**

**Description:** Comma separated identifiers of raw reads from runs analyzed in the scope of the metagenomic search. All datasets are searchable and downloadable by the identifier (https://www.ncbi.nlm.nih.gov/sra).

**File Name: Supplementary Data 4**

**Description:** Table containing all isolated minimal drz sequences from annotated bacteriophage genomes (see Supplementary Table 1) using the initial minimal drz search motif depicted in Fig. 2b (i). May contain a substantial amount of false positives.

**File Name: Supplementary Data 5**

**Description:** Table containing open reading frame annotations of phage genomes which contain at least one Θrz. More specific information is provided when available.

**File Name: Supplementary Data 6**

**Description:** Table containing all oligonucleotides used in this study including names, sequences, and short descriptions.
