# Supplementary material for: Identification of HDV-like theta ribozymes involved in tRNA-based recoding of gut bacteriophages
Source: Nat Commun. 2024 Feb 20;15:1559. doi: 10.1038/s41467-024-45653-w (PMC10879173; doi:10.1038/s41467-024-45653-w)
Supplement: Supplementary file 10 — Reporting Summary [file 41467_2024_45653_MOESM10_ESM.pdf]

nature portfolio

Corresponding author(s): Roland K.O. Sigel, Christian von Mering

Last updated by author(s): Dec 19, 2023

Reporting Summary

Nature Portfolio wishes to improve the reproducibility of the work that we publish. This form provides structure for consistency and transparency in reporting. For further information on Nature Portfolio policies, see our Editorial Policies and the Editorial Policy Checklist.

Statistics

For all statistical analyses, confirm that the following items are present in the figure legend, table legend, main text, or Methods section.

n/a	Confirmed	
☐	☒	The exact sample size (*n*) for each experimental group/condition, given as a discrete number and unit of measurement
☒	☐	A statement on whether measurements were taken from distinct samples or whether the same sample was measured repeatedly
☒	☐	The statistical test(s) used AND whether they are one- or two-sided<br>*Only common tests should be described solely by name; describe more complex techniques in the Methods section.*
☒	☐	A description of all covariates tested
☒	☐	A description of any assumptions or corrections, such as tests of normality and adjustment for multiple comparisons
☐	☒	A full description of the statistical parameters including central tendency (e.g. means) or other basic estimates (e.g. regression coefficient) AND variation (e.g. standard deviation) or associated estimates of uncertainty (e.g. confidence intervals)
☒	☐	For null hypothesis testing, the test statistic (e.g. *F*, *t*, *r*) with confidence intervals, effect sizes, degrees of freedom and *P* value noted<br>*Give P values as exact values whenever suitable.*
☒	☐	For Bayesian analysis, information on the choice of priors and Markov chain Monte Carlo settings
☒	☐	For hierarchical and complex designs, identification of the appropriate level for tests and full reporting of outcomes
☒	☐	Estimates of effect sizes (e.g. Cohen's *d*, Pearson's *r*), indicating how they were calculated

Our web collection on statistics for biologists contains articles on many of the points above.

Software and code

Policy information about availability of computer code

Data collection	No software was used for data collection.
Data analysis	All custom code is available under https://github.com/lukasmalfi/theta_ribozymes.<br>The following existing/external software was used:<br>genomad v1.3.3, RNArobo v2.1.0, tRNAscan-SE v2.0.9, Python v.3.7.6, Prodigal v2.6.3, OriginPro Version 2022, WordCloud v1.5.0, R2R v1.0.6, CorelDRAW X7 v17.1.0.572, pandas v1.0.3, NumPy v.1.18.1, Jupyterlab v3.5.0

For manuscripts utilizing custom algorithms or software that are central to the research but not yet described in published literature, software must be made available to editors and reviewers. We strongly encourage code deposition in a community repository (e.g. GitHub). See the Nature Portfolio guidelines for submitting code & software for further information.

Data

Policy information about availability of data

All manuscripts must include a data availability statement. This statement should provide the following information, where applicable:

- Accession codes, unique identifiers, or web links for publicly available datasets
- A description of any restrictions on data availability
- For clinical datasets or third party data, please ensure that the statement adheres to our policy

All data and intermediate results required to reproduce the study have been deposited in Zenodo accessible under https://zenodo.org/records/10299930. The viral

databases from open sources were obtained from the following links:
https://zenodo.org/record/4776317
http://ftp.ebi.ac.uk/pub/databases/metagenomics/genome_sets/gut_phage_database/
http://www.virusite.org/index.php?nav=download
https://zenodo.org/record/6410225
https://portal.nersc.gov/MGV
https://github.com/RChGO/OVD/
https://datacommons.cyverse.org/browse/iplant/home/shared/iVirus/GOV2.0
https://genome.jgi.doe.gov/portal/IMG_VR/IMG_VR.home.html
The bacterial genomes from ProGenomes3 can be downloaded here:
https://progenomes.embl.de/data/repGenomes/progenomes3.contigs.representatives.fasta.bz2
Source data are provided with this paper.

Research involving human participants, their data, or biological material

Policy information about studies with human participants or human data. See also policy information about sex, gender (identity/presentation), and sexual orientation and race, ethnicity and racism.

Reporting on sex and gender	NA
Reporting on race, ethnicity, or other socially relevant groupings	NA
Population characteristics	NA
Recruitment	NA
Ethics oversight	NA

Note that full information on the approval of the study protocol must also be provided in the manuscript.

Field-specific reporting

Please select the one below that is the best fit for your research. If you are not sure, read the appropriate sections before making your selection.

☒ Life sciences ☐ Behavioural & social sciences ☐ Ecological, evolutionary & environmental sciences

For a reference copy of the document with all sections, see nature.com/documents/nr-reporting-summary-flat.pdf

Life sciences study design

All studies must disclose on these points even when the disclosure is negative.

Sample size	No sample size was initially calculated. All available data was used for analysis.
Data exclusions	No data was excluded.
Replication	All experiments were performed in three independent replicates. Each attempt was successful. All three replicates are shown in the figures and listed in the supplementary tables.
Randomization	Randomization was not applicable in this study as all experiments were performed in vitro with no relevant experimental groups.
Blinding	Blinding was not pertinent to our study because it did not include any animals and/or human research participants.

Reporting for specific materials, systems and methods

We require information from authors about some types of materials, experimental systems and methods used in many studies. Here, indicate whether each material, system or method listed is relevant to your study. If you are not sure if a list item applies to your research, read the appropriate section before selecting a response.

Materials & experimental systems

n/a	Involved in the study
☒	☐ Antibodies
☒	☐ Eukaryotic cell lines
☒	☐ Palaeontology and archaeology
☒	☐ Animals and other organisms
☒	☐ Clinical data
☒	☐ Dual use research of concern
☒	☐ Plants

Methods

n/a	Involved in the study
☒	☐ ChIP-seq
☒	☐ Flow cytometry
☒	☐ MRI-based neuroimaging
